# Altered Cerebrospinal Fluid Concentrations of Hydrophobic and Hydrophilic Compounds in Early Stages of Multiple Sclerosis—Metabolic Profile Analyses

**DOI:** 10.1007/s12031-019-01336-6

**Published:** 2019-05-27

**Authors:** A. Podlecka-Piętowska, A. Kacka, B. Zakrzewska-Pniewska, M. Nojszewska, E. Zieminska, M. Chalimoniuk, B. Toczylowska

**Affiliations:** 1 Department of Neurology, Medical University of Warsaw, Zwirki i Wigury 61, 02-091 Warsaw, Poland; 2 Department of Anesthesiology, Medical University of Warsaw, Zwirki i Wigury 61, 02-091 Warsaw, Poland; 3 Department of Anesthesiology and Intensive Care, The Maria Skłodowska Curie Memorial Cancer Centre and Institute of Oncology, WK Roentgena 5, 02-781 Warsaw, Poland; 4 Department of Neurochemistry, Mossakowski Medical Research Centre Polish Academy of Sciences, Pawinskiego Str. 5, 02-107 Warsaw, Poland; 5 Department of Cellular Signaling, Mossakowski Medical Research Centre Polish Academy of Sciences, Pawinskiego Str. 5, 02-107 Warsaw, Poland; 6 Department of Tourism and Health in Biala Podlaska, Józef Piłsudski University of Physical Education in Warsaw, Marymoncka 34, 00-968 Warsaw, Poland; 7 Institute of Biocybernetics and Biomedical Engineering, Trojdena Str. 4, 02-109 Warsaw, Poland; 8 NMR Laboratory, Institute of Biochemistry and Biophysics, Pawinskiego Str. 5A, 02-107 Warsaw, Poland

**Keywords:** Multiple sclerosis, Inflammation, Metabolomics, NMR spectroscopy

## Abstract

The lack of a single predictive or diagnostic test in multiple sclerosis (MS) remains a major obstacle in the patient’s care. The aim of this study was to investigate metabolic profiles, especially lipids in cerebrospinal fluid (CSF) using ^1^H-NMR spectroscopy and metabolomics analysis to discriminate MS patient group from the control ones. In this study, 19 MS patients and 19 controls, without neurological problems, patients were enrolled. To obtain the CSF metabolic profiles, NMR spectroscopy was used. Hydrophilic and hydrophobic compounds were analyzed using univariate and multivariate supervised analysis orthogonal partial least square discriminant analysis (OPLS-DA). Targeted OPLS-DA analysis of 32 hydrophilic and 17 hydrophobic compounds obtained 9 hydrophilic metabolites and 8 lipid functional groups which had the highest contribution to patient’s group separation. Lower concentrations of CSF hydrophilic and hydrophobic compounds were observed in MS patients as compared to control group. Acetone, choline, urea, 1,3-dimethylurate, creatinine, isoleucine, myo-inositol, leucine, and 3-OH butyrate; saturated and monounsaturated acyl groups of ω–9, ω–7, ω–6, ω–3, and fatty acid, triglycerides, 1,3-DG, 1-MG, and unassigned component signal at 3.33 ppm were the most important signal compounds in group separation. Analysis of metabolic profile of raw CSF and their lipid extract shows decreased levels of many compounds and led to the conclusion that MS patients could have a disturbance in many metabolic pathways perhaps leading to the decreased level of acetyl-CoA and/or inflammation. CSF metabolic profile analyses could be used as a fingerprint for early MS diagnosis.

## Introduction

Multiple sclerosis (MS) is a chronic autoimmune disease that affects the central nervous system (CNS) characterized by demyelination and simultaneous axonal and neuronal degeneration that occurs from the earliest clinical stage of the disease (Campbell et al. 2011). Although the causes of MS are not completely known, it is notorious that this disease is characterized by heterogeneous and multifaceted mechanisms involving oxidative damage (Fischer et al. 2013), increased inflammation (Lubina-Dabrowska et al. 2017), and mitochondrial injury (Campbell and Mahad 2012). At the places of damaged axons and oligodendrocytes, the changes in the inflammation occur which cause the formation of active and inactive demyelinating plaques in the brain (Brosnan et al. 1996). In the demyelinating plaques, activated astrocytes, microglia, T cells, and macrophages occur which in turn secrete pro-inflammatory factors such as cytokines (IL-6, IL-1β, TNFα, INFγ), free oxygen radicals (ROS), and nitric oxide (NO) (Brosnan et al. 1996; DeGroot et al. 1997). All the above-mentioned pro-inflammatory mediators are found to be elevated in the cerebral cortex, cerebrospinal fluid (CSF) as well as in the serum in MS patients. In 90% of patients with MS, local disturbances of B cells’ response elicit presence of oligoclonal bands in CSF which have been proposed as a helpful biomarker for MS diagnosis and evaluation of treatment*.* Their presence discloses the intrathecal immunoglobulin G (IgG) synthesis (Bo et al. 1994)*.* However, recent studies have indicated a large number of controversies about the oligoclonal IgG bands’ role in MS (O'Connor et al. 2003). Currently, diagnosis of MS is based on clinical criteria including symptoms, magnetic resonance imaging (MRI), lumbar puncture to identify inflammatory proteins, and excluding other disorders (Zhou et al. 2016; Hunter 2016; Raphael et al. 2015). For many patients, diagnosis takes months, and the decision of introduction of MS therapy is delayed. The lack of a single predictive or diagnostic test in MS remains a major obstacle in the patient’s care. In recent years, there have been advances in molecular biology, cellular immunology, and “omics” (genomics, proteomics, metabolomics) which focus on exploring the processes underlying disease pathogenesis to provide a list of possible MS biomarkers (Kuhle et al. 2015; Poddighe et al. 2017).

In the last decade, advances in high-throughput approaches allowed development of proteomic and metabolomics studies in evaluating the association of genetic and phenotypic variability with disease sensitivity and therapy response. These considerations have more value in the case of MS, a multifactorial disease with high heterogeneity in clinical course, and treatment response.

Metabolomics concerns the identification and quantification of small endogenous molecules in a biological system. Because the metabolite represents substrates and the final products of physiological processes in a living organism, the profiling of the metabolome in tissues and biofluids offers an instantaneous molecular image of the phenotype. Among the several analytical techniques, HPLC, high-resolution mass spectrometry, and gas chromatography coupled with mass spectrometry are the most commonly used methods in the metabolomics filed (Kim et al. 2017; Del Boccio et al. 2016; Poisson et al. 2015; Dickens et al. 2015; Cha et al. 2015; Pieragostino et al. 2018). These techniques have been used to investigate MS in serum and CSF hydrophilic and hydrophobic compounds (Bhargava and Calabresi 2016; Cocco et al. 2016; Moussallieh et al. 2014; Reinke et al. 2014)*.* Nuclear magnetic resonance (NMR) spectroscopy is not often presented in CSF metabolomics of MS patients. In CSF studies of most research groups, other neurological disease (OND) patients have been used as the control group. The participation of metabolomics in the autoimmune process of MS has been examined in serum, but the role of lipids, especially in combination with amino acids, remains poorly defined. Lipids play a main dual role in MS, both as substrates of myelin and as mediators of inflammation.

The aim of this study was to investigate metabolic profiles, especially lipids in CSF using ^1^H-NMR spectroscopy and metabolomics analysis to discriminate the MS patient group from the control ones. Our studies focused on metabolic and lipid profiles of the same CSF sample and compared the results of MS patients to control, non-neurological problem patients. We hypothesized that disturbances in CSF metabolite profiles reflected the myelin degradation/regeneration during the inflammatory process of brain tissue in MS relapsing/remitting status.

## Materials and Methods

### Patients

In this study, 19 patients (13 females and 6 males) with MS according to the McDonald criteria of 2010 (Polman et al. 2011) were enrolled in the study in 2016–2017. Patients were admitted to the department of neurology for MS diagnosis. All patients underwent extensive neurologic evaluation; impairment and disability were measured using the Expanded Disability Status Scale (EDSS). None of them had a history of immunomodulatory or immunosuppressive therapy. All patients had MRI to show characteristic multiple lesions in the periventricular and subcortical white matter of the brain as well as gadolinium enhancements, a result of active demyelination. We excluded patients with any other chronic diseases: depression, diseases of liver, kidney, thyroid gland, and abnormalities in blood morphology. The analysis of CSF obtained from all the patients was done after setting the first diagnosis of MS. Based on the clinical data, all MS patients had an active disease status.

The control group of 19 patients (12 females and 7 males) was set from those who underwent minor vascular surgery or inguinal hernia repair under spinal anesthesia during the last 2 years and had not any neurological problems.

At the time of screening for trial inclusion, potential participants received thorough written and oral information on the purpose and duration of the study, as well as possible adverse events, and signed the informed consent. The study was designed in accordance with the Declaration of Helsinki. The study protocol was approved by the Hospital Bioethics Committee.

### Sample Preparation and Spectra Acquisition

Two milliliters of CSF were collected from lumbar puncture during anesthesia or clinical examination. All samples were centrifuged at room temperature at 15,000 rpm for 5 min, and the supernatants were frozen at − 80 °C until NMR analysis was performed. The pH was stabilized at 7.4 ± 0.2 using HCl. To achieve a stable lock signal, a 100 μl of D_2_O was added to each sample volume. 3-Trimethylsilyl propionic acid (TSP) with a final concentration in the sample of 1 mM was used as an internal reference compound for the normalization of all spectra, quantitative statistical analysis. Hydrophobic compounds were prepared according to the procedure described in our past publication (Zieminska et al. 2018).

All NMR spectra were acquired at 25 °C on a Varian Inova 400 (Varian Inc.) spectrometer. One pulse sequence was applied to hydrophilic and hydrophobic CSF compounds in ^1^H-NMR examinations. Settings for each measurement were for raw and CDCl_3_ samples: 512/128 transients, 12/5 s pulse repetition time, respectively. Zero-filling to 16 k data points, line broadening of 0.5, baseline and phase correction were applied to each spectrum using software implemented in the spectrometer. Signals were assigned according to our own reference database and literature information (Lutz et al. 1998; Wevers et al. 1995).

### Data Analysis

Quantities of metabolites were expressed as relative intensity. Spectra were both baseline and phase corrected and normalized to the TSP or CDCl_3_ rest signal prior to statistical analysis. For the statistical analysis of raw CSF samples and lipid spectra, 32 and 18 signals of the NMR spectrum were selected, respectively.

Univariate statistical analysis was performed for all data using the Mann-Whitney test followed by Tukey correction. A *P* value lower than 0.05 was considered as significant.

Multivariate statistical analysis was performed using supervised methods of orthogonal partial least square discriminant analysis (OPLS-DA). In the OPLS-DA modeling, the goodness of fit is reported as the cumulative score across all of the components R2cum—explained by the model and Q2cum—predicted by the model. OPLS-DA model was considered significant if R2cum and Q2cum were significantly larger than zero and was considered as good when both values were equal or greater than 0.5 (Bylesjo et al. 2008). The variable importance in the projection (VIP) value of each variable in the model was calculated to indicate its contribution to the classification. Those variables with VIP value greater than 1.0 were considered significantly different, and a larger VIP value of a variable represented a higher contribution to the discrimination between groups (Karp et al. 2005; Toczylowska et al. 2013). A receiver-operator characteristic (ROC) curve was created by plotting the true positive rate (TPR) versus the false positive rate (FPR = 1-TNR) at various threshold settings of the criterion parameter. As a quantitative measure of the classification success, the area under the ROC-AUC was calculated. Multivariate analysis, OPLS-DA, was performed using the software package SIMCA-P (Version 14, MKS Umetrics, Sweden) (Ellis et al. 2007).

## Results

The MS group of patients had an average age of 34.2 ± 9.6, with median 2.5 years of disease (1–15 years), median EDSS value was 1.75 (0–3.5), the median number of relapses before admission was 1 (0–3) (Table 1). All the patients had been diagnosed with at least one active lesion in the brain (9 out of 19) and/or in the spinal cord (12 out of 19). Routine CSF examinations showed cytosis as well as oligoclonal IgG bands in all patients.

**Table 1:** Clinical parameters of control and MS groups

Parameter	Control	MS
N (F/M)	19 (12/7)	19 (13/6)
Age	46.2 ± 12.7	34.2 ± 9.6
EDSS value (median/min/max)	–	1.75 (0–3.5)
Number of relapses (median (min–max))	–	1 (0–3)
Cytosis (number/mm^3^)	1.63 ± 0.33	3.74 ± 2.83*(*p* = 0.009)
Protein (mg%)	26.44 ± 3.97	39.12 ± 10.82* (*p* < 0.001)
Glucose (mg/dL)	57.89 ± 7.5	57.50 ± 11.46

*Significant differences between groups

### The Concentrations of CSF Hydrophilic Compounds

All 32 hydrophilic compounds selected for the identification of potential differences in biochemical composition caused by neuronal death processes, the obtained *P* value for univariate analysis, and VIP value > 1 are presented in Table 2.

**Table 2:** Direction of changes and statistical significance of analyzed hydrophilic compounds of CSF fluid for MS vs control group

Compound		MS vs control	*P* value	VIP value	Published results
ppm	Assignment				
8.46	Formate	↓	0.550		
7.73	Hist	↓	0.261		
7.39	Phe	↓	0.389		
6.86	Tyr	↑	0.872		
5.76	Urea	↓	0.006	1.57	↓ (Sinclair et al. 2010; Koneczny et al. 2014)
4.12	Lactate	↓	0.457		
4.07	Myo-inositol	↓	0.015	1.13	↓ (Sinclair et al. 2010)
4.01	Betaine	↓	0.804		
3.52	Gly	↓	0.782		
3.36	1,3 dimethylurate	↓	0.358	1.45	↓ (Sinclair et al. 2010)
3.31	Cysteine	↓	0.274		
3.25	Glucose	↓	0.693		
3.21	Choline	↓	0.008	1.82	↓ (Sinclair et al. 2010)
3.16	Citrulline	↓	0.166		
3.05	Creatinine	↓	0.007	1.19	↓ (Sinclair et al. 2010)
3.04	Creatine	↓	0.140		
2.68	Citrate	↓	0.569		
2.42	Gln	↓	0.872		
2.38	Pyruvate	↓	0.550		
2.28	Acetoacetate	↓	0.530		
2.11	Methionine	↓	0.550		
2.24	Acetone	↓	0.001	2.10	↓ (Sinclair et al. 2010)
2.13	Gln	↓	0.362		
2.03	N-acetyl NH_3_ group	↓	0.942		
1.93	Acetate	↓	0.942		
1.72	Lys	↓	0.872		
1.47	Ala	↓	0.849		
1.34	Lactate	↓	0.511		
1.21	3-OH-butyrate	↓	0.157	1.12	↓ (Sinclair et al. 2010)
1.03	Val	↓	0.257		
0.96	Ile	↓	0.140	1.09	↓ (Sinclair et al. 2010)
0.9	Leu	↓	0.052	1.18	↓ (Sinclair et al. 2010)

**Fig. 1:**
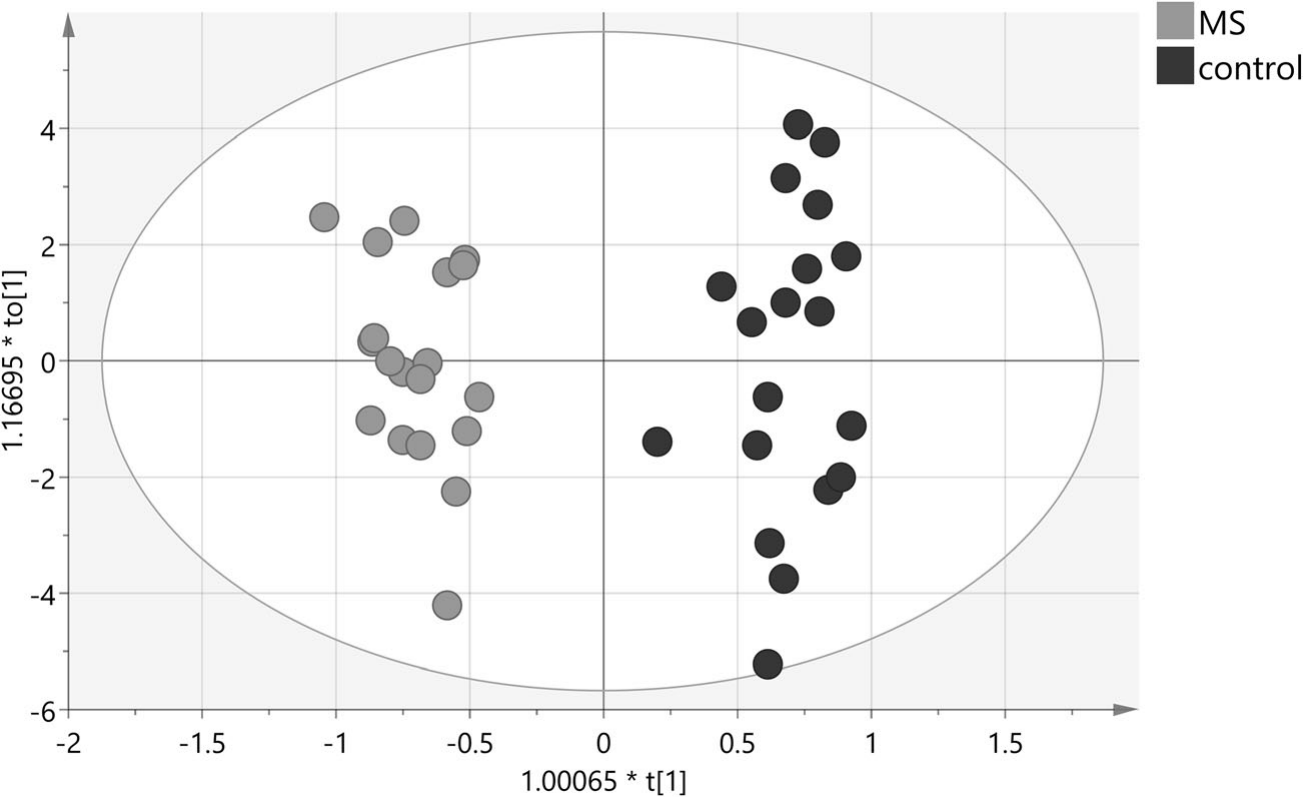
The score plot of the two-component OPLS-DA model for hydrophilic compounds of NMR data; to[1] represents within class variation in the first orthogonal component, whereas t[1] represents between class variation in the first predictive component. Ellipse represents Hotelling T2 with 95% confidence in score plots

**Fig. 2:**
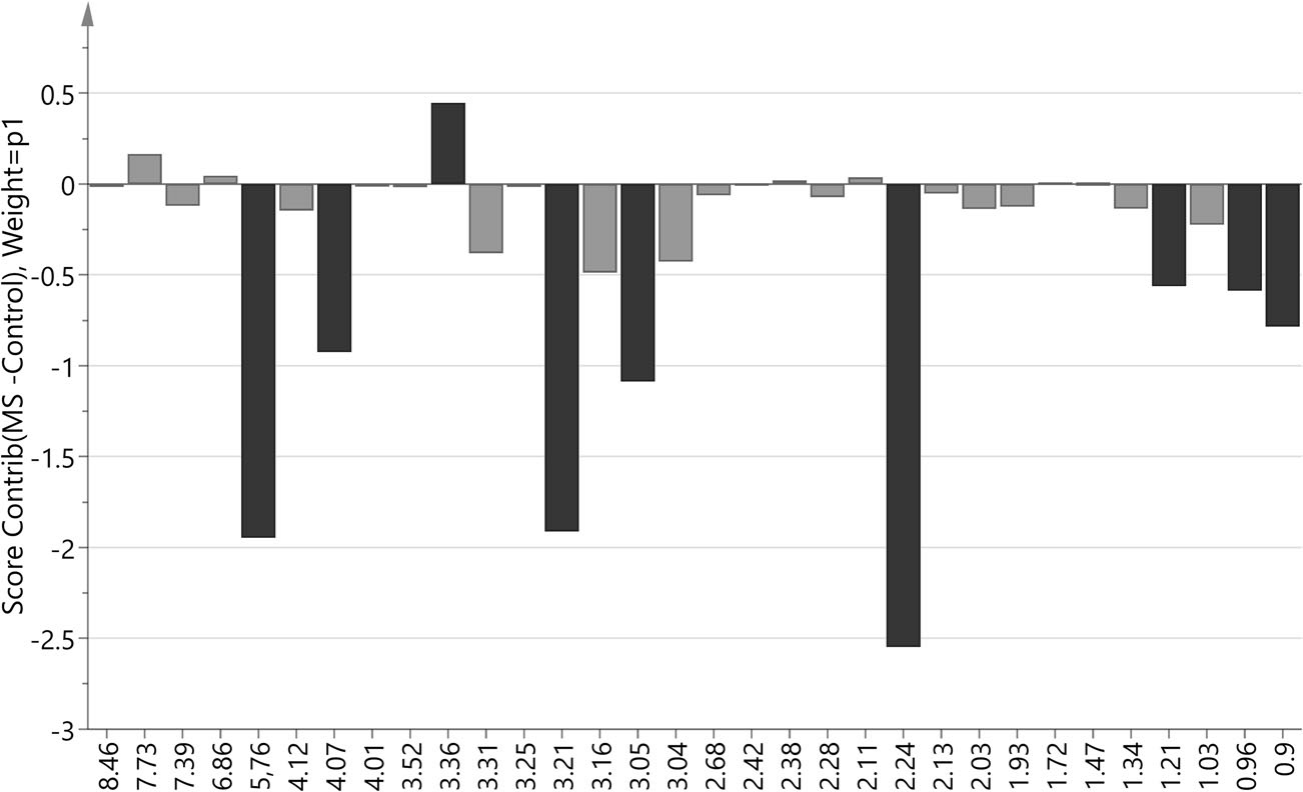
Score contribution for hydrophilic compounds for MS group versus control group. Black color indicated metabolites with VIP > 1

**Fig. 3:**
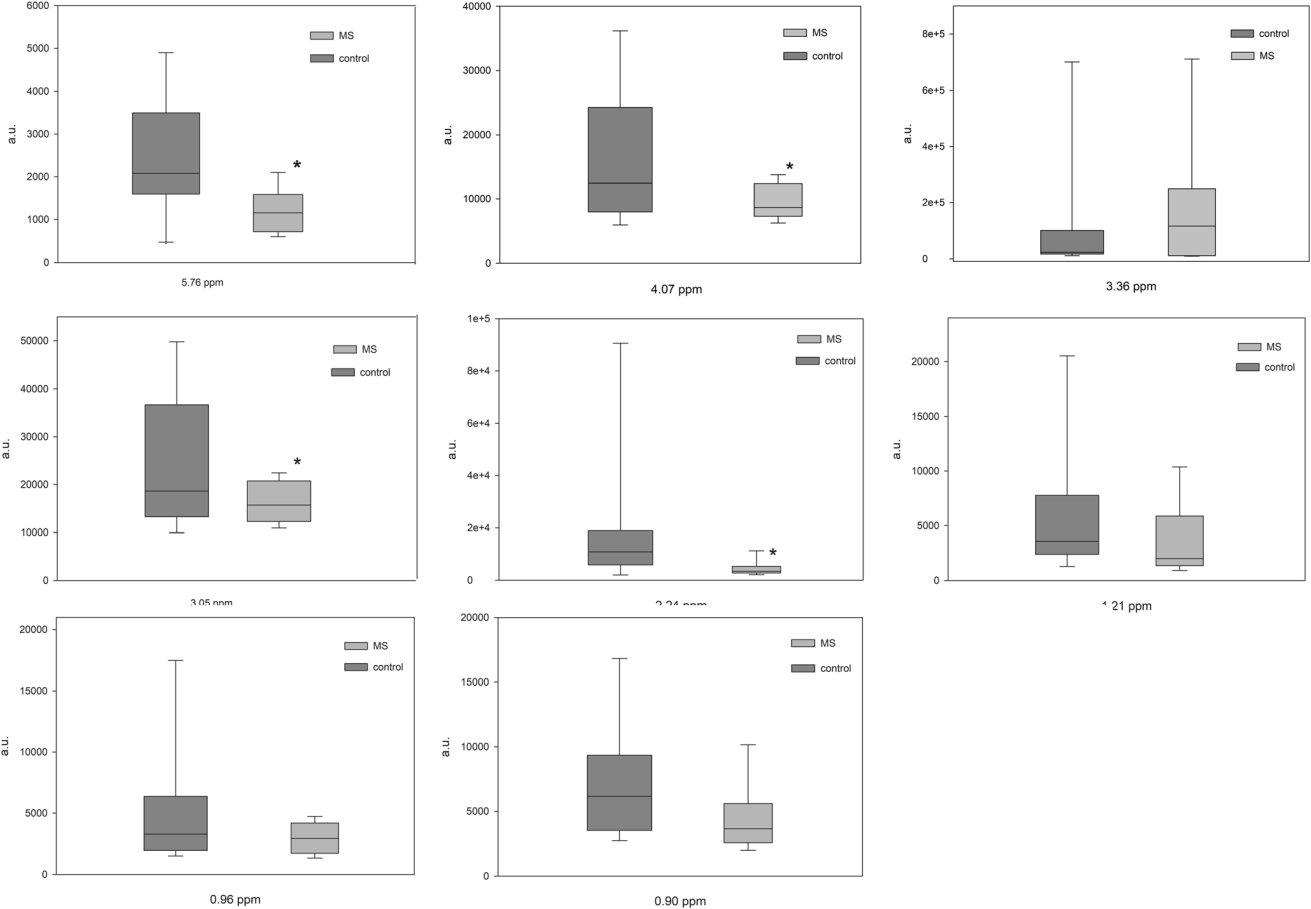
Box and Whisker plot of hydrophilic compounds of VIP > 1 compounds. “*” indicated differences with *p* < 0.05 (Mann-Whitney test)

**Fig. 4:**
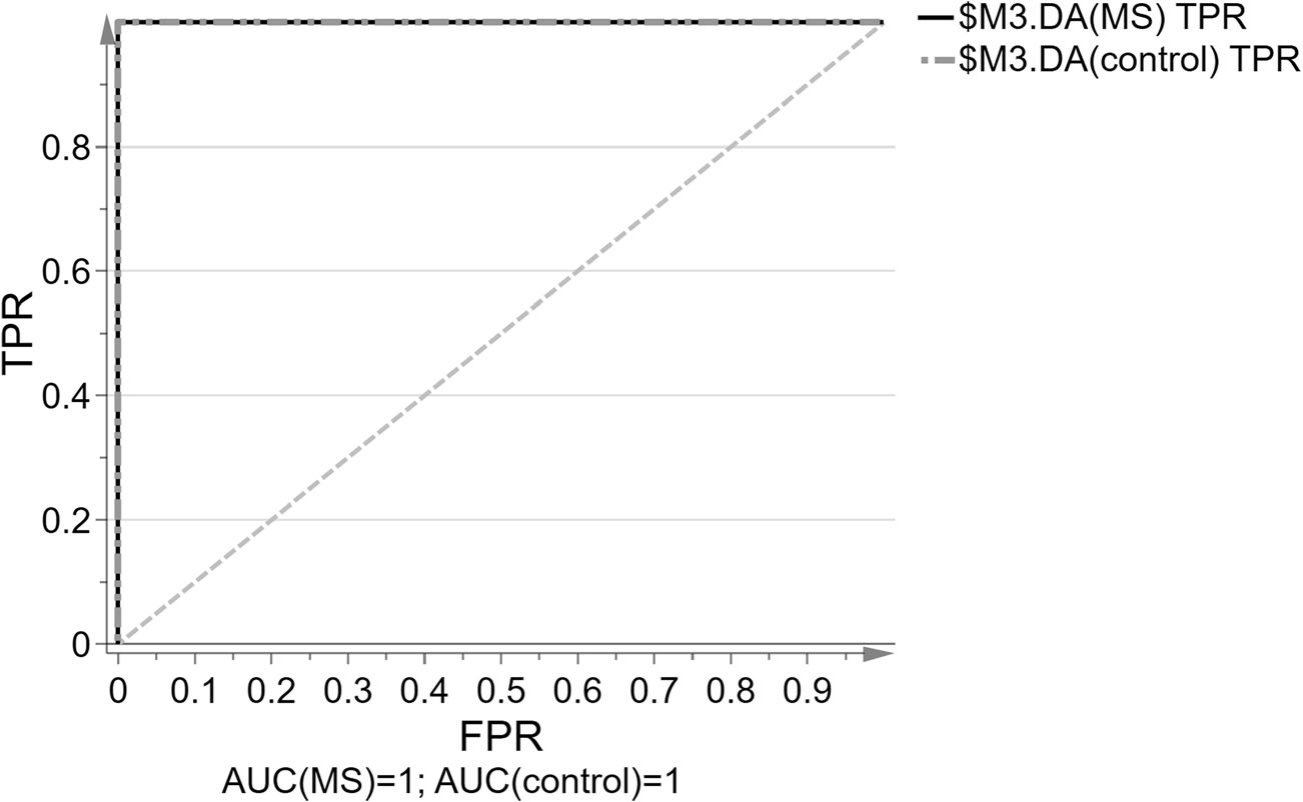
The ROC curve of the true positive rate (TPR) versus the false positive rate (FPR = 1-TNR) and ROC-AUC values for both groups as quantitative measure of the classification success for hydrophilic compounds for the OPLS-DA model; sensitivity: 100%, 95% CI = 82.35% to 100.00%; specificity: 100%, 95% CI = 82.35% to 100.00%

The multivariate OPLS-DA modeling was employed using the knowledge of patient’s classification. The best OPLS-DA model consisted of one predictive and seven orthogonal components (R2cum = 0.948, Q2cum = 0.703). In this model, 100% of all patients were classified correctly according to their groups (Fig. 1). The most important parameters (VIP > 1) that contributed to class separation were the NMR signals from acetone, choline, urea, 1,3-dimethylurate, creatinine, isoleucine, myo-inositol, leucine, and 3-OH-butyrate. The model was tested for validity by applying the analysis of variance to cross-validated predictive residuals (*F* test, *P* = 0.004). In the MS group, all metabolites had lower concentrations as compared to the control group (Figs. 2 and 3). A receiver-operator characteristic (ROC) generated from the ratio of the sensitivity to 1—selectivity resulted in an area under the curve of 1.0 for both, control and MS groups, which was the perfect classification (Fig. 4).

### The Concentration of CSF Hydrophobic Compounds

**Fig. 5:**
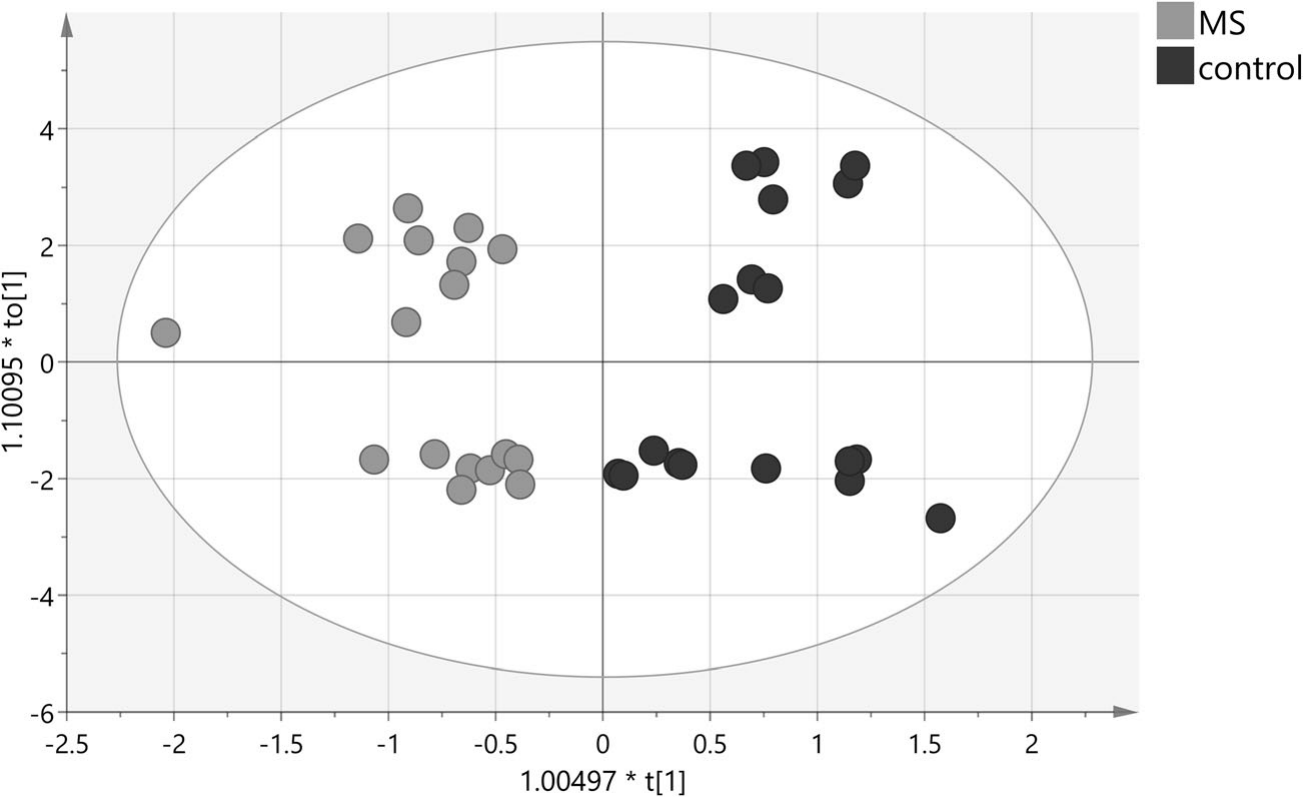
The score plot of the two-component OPLS-DA model for hydrophobic compounds for NMR data; to[1] represents within class variation in the first orthogonal component, whereas t[1] represents between class variation in the first predictive component. Ellipse represents Hotelling T2 with 95% confidence in score plots

**Fig. 6:**
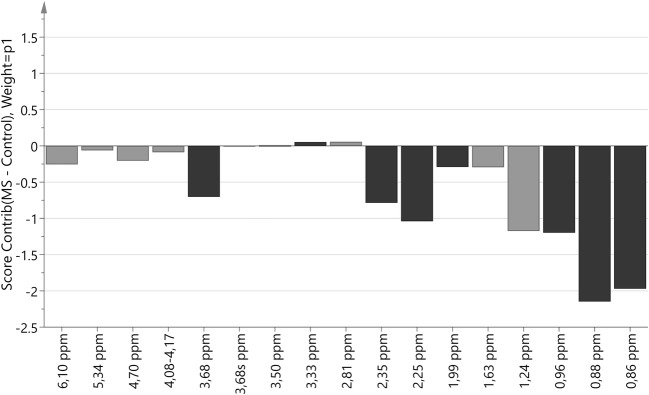
Score contribution for hydrophobic compounds for MS group versus control group. Black color indicated metabolites with VIP > 1

**Fig. 7:**
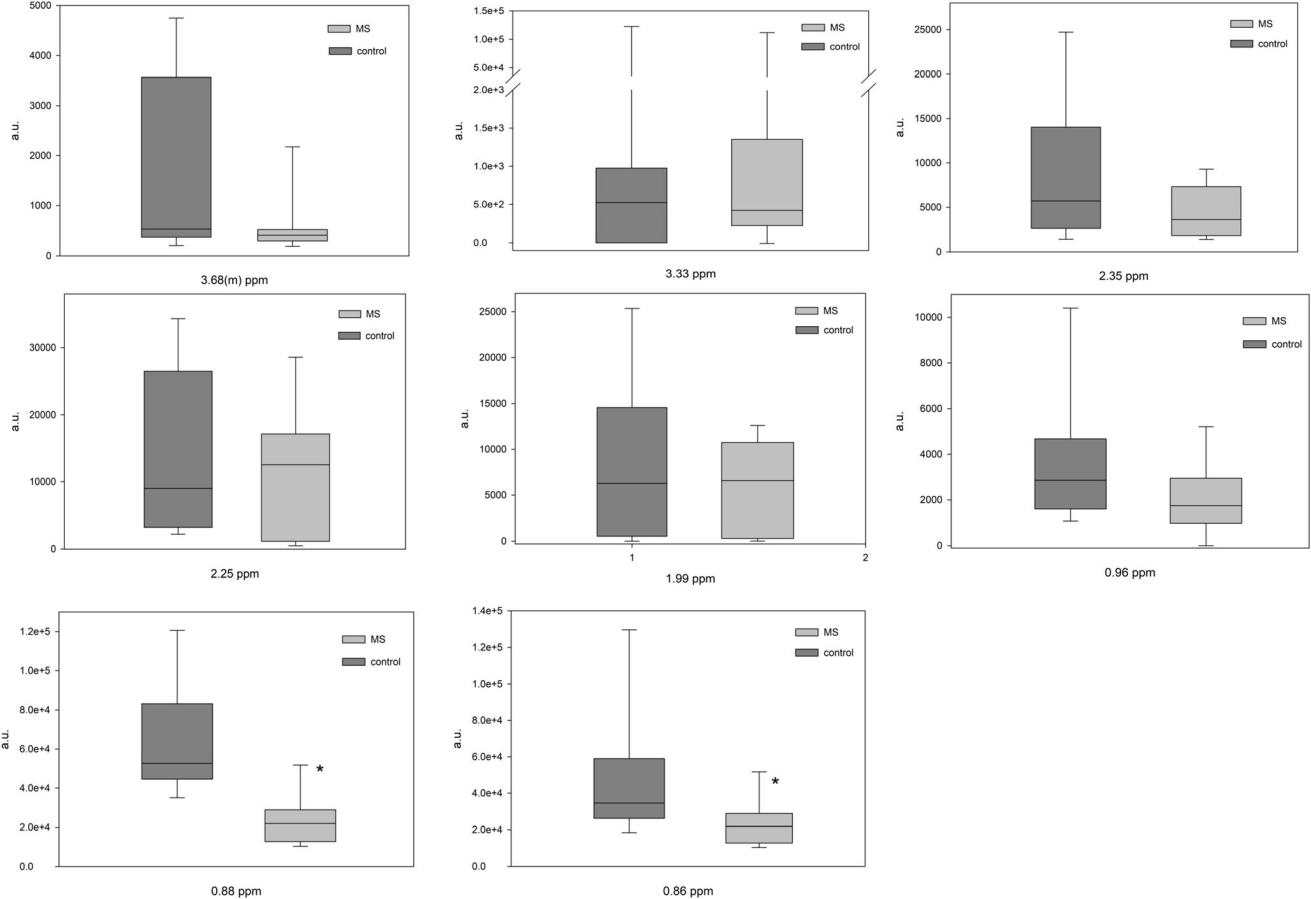
Box and Whisker plot for hydrophobic compounds of VIP > 1 NMR signals. “*” indicated differences with *p* < 0.05 (Mann-Whitney test)

**Fig. 8:**
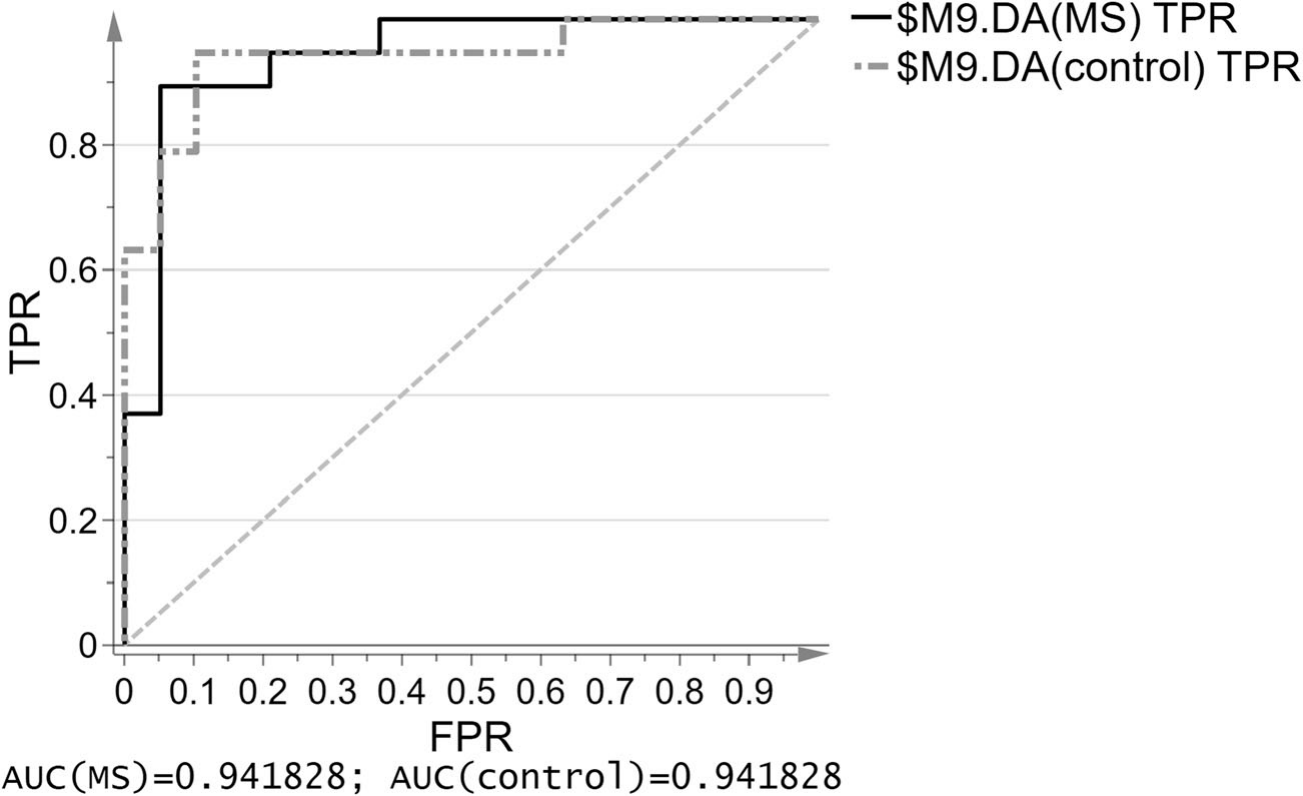
The ROC curve of the true positive rate (TPR) versus the false positive rate (FPR = 1-TNR) and ROC-AUC values for both groups as quantitative measure of the classification success for hydrophobic compounds for the OPLS-DA model; sensitivity: 92.3%, 95% CI = 63.97% to 99.81%; specificity: 72%, 95% CI = 50.61% to 87.93%

Seventeen lipid compound functional groups from proton NMR signals are presented in Table 3 with *P* value of univariate analysis and VIP > 1 values. Discriminant analysis, OPLS-DA, of the lipid compound data allowed building a model (Fig. 5). The model consisted of one predictive and four orthogonal components (R2cum = 0.787, Q2cum = 0.528). In this model, 92% of all patients were classified correctly according to their groups (12 out of 19 in the MS group and 18 out of 19 in the control group). The most important parameters (VIP > 1) that contributed to class separation were the signals from –CH_3_– saturated, monounsaturated ω–9 and/or ω–7 acyl groups and fatty acid (FA) (0.86 ppm), –CH_3_– unsaturated ω–6 acyl groups and FA (0.88 ppm), –CH_3_ unsaturated ω–3 acyl groups and FA (0.96 ppm), –CH_2_–CH=CH– acyl groups and FA (1.99 ppm), –OCO–CH_2_–acyl groups in triglyceride (TG) (2.25 ppm), –OCO–H_2_–,–COOH–CH_2_– acyl groups in 1,3-DG, 1-MG, and FA (2.35 ppm), ROCH_2_–CHOH–CH_2_OH glyceryl group in 1-MG (3.68 ppm) and unassigned signal at 3.33 ppm. The model was tested for validity by applying the analysis of variance to cross-validated predictive residuals (*F* test, *P* = 0.02). In the MS group, all compounds had a lower concentration as compared to control group except unassigned functional group signal (singlet) at 3.68 ppm and =HC–CH_2_–CH= diunsaturated ω-6 acyl groups and FA that had a higher concentration as compared to control group (Figs. 6 and 7). A receiver-operator characteristic (ROC) generated from the ratio of the sensitivity to 1-selectivity resulted in an area under the curve of 0.94 for both, control and MS groups, which was the almost perfect classification (Fig. 8).

**Table 3:** Direction of changes and statistical significance of analyzed hydrophobic functional groups of CSF fluid for MS vs control group

Compound		MS vs control	*P* value	VIP value	Publish results
ppm	Functional group				
6.10	Estriol (–HC(2,4)=)	↓	0.378		
5.34	–HC = CH– in FA	↓	0.608		
4.70	Unassigned 1	↓	0.222		
4.17–4.08	ROCH_2_-CHOH-CH_2_OR′ in glyceryl group of 1,3-DG	↓	0.457		
3.68_m_	ROCH_2_–CHOH–CH_2_O in glyceryl group of 1-MG	↓	0.107	1.09	↓ (Pietrocola et al. 2015)
3.68_s_	Unassigned 2	↑	0.734		
3.50	Pregnenolon	↓	0.522		
3.33	Unassigned 3	↓	0.854	1.04	
2.81	=HC–CH_2_–CH = in acyl groups of diunsaturated ω-6 and FA	↑	0.861		
2.35	–OCO–CH_2_–, –COOH–CH_2_– in acyl groups of 1,3-DG, 1-MG and FA	↓	0.082	1.04	↓ (Pietrocola et al. 2015)
2.25	–OCO–CH_2_– in acyl groups of FA	↓	0.243	1.34	↓ (Pietrocola et al. 2015)
1.99	–CH_2_–CH=CH– in acyl groups of FA	↓	0.492	1.08	↓ (Pietrocola et al. 2015)
1.63	–OCO–CH_2_–CH_2_– in acyl groups of 1,3-DG, 1-MG and FA	↓	0.249		
1.24	–(CH_2_)_n_– in acyl groups of FA	↓	0.013		
0.96	–CH_3_ in acyl groups of unsaturated ω-3/FA	↓	0.060	1.12	↓ (Pietrocola et al. 2015)
0.88	–CH in acyl groups of unsaturated ω-6/FA	↓	0.001	1.28	↓ (Pietrocola et al. 2015)
0.86	–CH_3_ in acyl groups of saturated, monounsaturated ω-9 and/or ω-7 and FA	↓	0.003	1.34	↓ (Pietrocola et al. 2015)

NMR signal: *s* singlet, *m* multiplet

## Discussion

In this study, investigators performed metabolic and lipids profiling using ^1^H-NMR spectroscopy of CSF samples from the early stage of MS to define the differences between MS and control-matched subjects. This approach can detect a number of endogenous metabolites that influence the condition to change an organism in MS. Thus, identification of the important metabolites allows evaluation of potential pathways characterizing an early stage of the disease. The model created in this study revealed differences in the metabolites and lipids of CSF in the two groups. Therefore, the authors looked at a possible correlation in the metabolic profiles of hydrophilic and hydrophobic compounds with clinical parameters to study their relationship with pathology. The most important problem in comparing the results of different research groups is the use of different control groups. Most groups have used OND patients as the control group. In our study, we used non-neurological problem patients as the control group.

By targeted analysis of OPLS-DA, 32 hydrophilic metabolites and 17 hydrophobic compound functional groups were studied. Investigators obtained nine NMR signals associated with hydrophilic metabolites and eight NMR signals associated with lipid functional groups which had the highest contribution to patient’s group separation.

Lower concentrations of CSF hydrophilic compounds were observed in MS patients as compared to control group. Univariate analysis indicated significant differences in urea, myo-inositol, 1,3-dimethylurate, choline, creatinine, and acetone concentrations while OPLS-DA indicated the low significant concentration of acetone, choline, urea, 1,3-dimethylurate, creatinine, isoleucine, myo-inositol, leucine, and 3-OH butyrate as in other group studies (Sinclair et al. 2010). Noga et al. (2012) have demonstrated a significant change in amino acid metabolism in CSF during EAE. They found the decreased level of metabolites related to pathways including NO synthesis, energy metabolism, polyamine synthesis, and levels of endogenous antioxidants. Sinclair et al. (2010) have shown a similar change in the metabolite levels in CSF as was observed in the present study. The metabolic changes of MS may be related to altered energy metabolism and FA biosynthesis in the brain. Down-regulation of citrated and acetate may support disruption of TCA cycle through pyruvate pathway. This was confirmed by the reduced metabolism of lipid compounds in CSF in this study.

Acetone is an end product of ketosis, a metabolic state that produces ketone bodies for use as another fuel for the brain. Both the levels of 3-hydroxybutyrate (non-significant *P* < 0.157) and acetone were altered in patients as compared to the control subjects in this analysis. The significant decrease of acetone in CSF might imply that the decreased flux from acetyl-CoA into acetoacetyl-CoA resulted in lower production of acetone or a reduced efflux through the blood-brain barrier (BBB) and higher consumption in the brain. Interestingly, the expression level of GULT 1, a major glucose transporter in the BBB, is down-regulated in the brain lesion of MS patients. Consistently, the observations for disturbed energy generation in CNS diseases including MS have been reported: mitochondrial dysfunctions detected in MS lesions as well as OND (Joseph et al. 2009; Ronowska et al. 2018).

On the analysis of lipid levels of CSF, especially saturated and unsaturated FAs, we observed their decrease in MS patient group as compared to the control object group. FAs are structural compounds that are components for cell membrane building. They are synthesized from cytosol compounds and acetyl-CoA from cytosol or mitochondria. In NMR experiments, used in these studies, it is not possible to measure very low concentrations of acetyl-CoA because it is below the sensitivity level of this method. However, we can measure the levels of compounds which are the products of cycles with acetyl-CoA participation which is necessary to synthesize FA and ketone bodies. We observed the decrease of FA and PUFA levels which could be the effect of altered level of acetyl-CoA (Pietrocola et al. 2015). In this study, we observed the decreased levels of ketone bodies (acetone, 3-OH butyric acid) that are indirect metabolites of decarboxylation of acetoacetate in the lipid degradation process. It could be the result of glycolysis cycle where acetyl-CoA participates. Low level of ketone bodies inhibits dopamine secretion (Cornille et al. 2010).

In our study, we observed altered choline and urea levels in the MS group as compared to the control group. Choline in the brain is an essential component in cholesterol and other lipids metabolism. The decrease of choline level influences their metabolism, further reducing their levels, similar to what we observed in our study. It also influences the so-called integrity and fluidity of the cell membrane (Zhong et al. 2014). Choline is a substrate for the synthesis of acetylcholine (ACh), excitation neurotransmitter. All immune cells have receptors for many neurotransmitters including ACh and neuropeptides. Immune cells stimulate the immune and brain system, and thus, disturbances in ACh influence their function. This may result in immunological disturbances including possible autoimmune reaction. The level of urea, the end compound of protein degradation cycle, is decreased in neuromuscular diseases, e.g., myasthenia gravis and dystrophies (Koneczny et al. 2014).

Another low-level compound in MS group is myo-inositol. It is partially synthesized in the brain and is an important part of glycolipids and cell membrane building compounds. It sensitizes serotonin and GABA receptors (Balla 2013).

We also observed decreased CSF creatine level in the MS patient group. Creatine takes part in phosphocreatine metabolism which in turn is the main energetic substrate for cells. Their decrease indicates energetic disturbances in neuronal cells. Brewer (Brewer and Wallimann 2000) demonstrated the neuroprotective role of creatine in their studies on rat hippocampus. The decrease of creatine level and thus energetic deficiency can correlate with MS neuronal deficiencies. This hypothesis was supported by the observed decreased levels of two ketogenic amino acids: leucine and isoleucine. Metabolism of ketogenic amino acids leads to the formation of acetyl-CoA or acetoacetyl-CoA. If they will not be completely utilized in the TCA cycle, the rest may undergo ketogenesis.

The most relevant results of our studies were the detection of an altered level of specific hydrophobic functional groups in the MS group compared to the control subjects. In particular, we found a significant decrease level in the MS group of saturated, monounsaturated acyl groups of ω–9 and/or ω–7, ω–6, ω–3, and FA, TG, 1,3-DG, 1-MG, glycerol group in 1-MG, and unassigned component signal at 3.33 ppm.

Pieragostino (Pieragostino et al. 2015) has analyzed the hydrophobic metabolites of MS and OND patients’ CSF using MALDI-TOF mass spectrometry. This method needs special preparation of the samples (internal labeled standards) and therefore, is more complicated and more expensive. Our results differed from those obtained by the Pieragostino study because different control groups were examined (healthy vs. OND). The most relevant result from their studies is the altered levels of specific phospholipids in the MS group compared to the OND group. In particular, they reported a significantly increased level of lysophosphocholine (LPC) (18:1), (18:0), lysophosphoinositol (16:0) in the MS patients. LPCs are well correlated to Link index (also known as IgG Index) which is the parameter indicating high levels of intrathecal IgG synthesis. Intrathecal IgG synthesis is a common event in part of MS. In our patients, IgG index was elevated in CSF. We also obtained the down expression of lipid compounds in the CSF of MS group, a cerebral component suggestive for a possible function of these lipids as candidate biomarkers, reflecting intrathecal synthesis IgG and CNS inflammation.

Clinical and MRI studies indicate that axonal damage predominantly appears in the early MS and develops as a consequence of inflammatory process (Bendfeldt et al. 2009), leading to the most numerous (~ 85% of cases) relapsing-remitting form of the disease (Weiner 2008). Elevated levels of aforementioned pro-inflammatory cytokines and lipid peroxidation in the plasma, cerebrospinal cord, and brain cortex have been found in the patients with MS (Gonsette 2008; Keller and Mattson 1998); a positive correlation has been found between their levels and disease’s activity and severity (Sharief 1991; Navikas and Link 1996). Gonzalo et al. (Gonzalo et al. 2012) performed targeted lipidome analyses comprising several oxidized phospholipids, lipid peroxidation-derived aldehydes, oxysterols, and oxidized lipids. The results confirmed the presence of aldehyde in agreement with data by Negre-Salvayre (Negre-Salvayre et al. 2010) in human MS showing increased lipid peroxidation in serum. Lipid peroxidation can exert part of its pathological properties through modification of the protein. Lipids are the major species of cell membranes and removal of one of the FA results in the increase of lysophospholipids (LPL) usually through the enzymatic action of a phospholipase A2 (PLA2). Several studies reported the altered levels of phospholipase (PL) in neurodegenerative diseases concluding that secretary PLA2 activity in CSF might serve as a valuable biomarker of inflammation as demonstrated in Alzheimer’s disease (Chalbot et al. 2009). In EAE model of SM, the blockage of PLA2 is highly efficacious in the amelioration of the disease courses probably by reducing T cell proliferation, pro-inflammatory cytokine production preventing activation of CNS microglia, and increasing myelin protein levels (Siritho and Freedman 2009).

## Conclusion

The use of ^1^H-NMR spectroscopy was driven to obtain many compounds in CSF and to be able to carry out the identification of unknown compounds as well as to apply an easy robust methodology to be transferred into clinical practice. Analysis of metabolic profile of raw CSF and their lipid extract showed decreased levels of many compounds and led to the conclusion that MS patients could have a disturbance in FA synthesis as well as in other metabolic pathways perhaps leading to the decreased levels of acetyl-CoA. This, in turn, could reflect the disturbance processes of myelin regeneration and influence the neurotransmission processes (excitation/inhibition) due to energetic disturbances. Changes in the concentration of the compounds were detected using NMR-based metabolite profiles, and direction of those changes was in agreement with results of studies done by other research groups. Therefore, CSF metabolite profile analyses could be used as a fingerprint for early MS diagnosis.
